# Inactivation kinetics of *Listeria monocytogenes* and *Salmonella enterica* on specialty mushroom garnishes based on ramen soup broth temperature

**DOI:** 10.3389/fmicb.2024.1485398

**Published:** 2024-12-04

**Authors:** Joelle K. Salazar, Megan L. Fay, Gregory Fleischman, Bashayer A. Khouja, Diana S. Stewart, David T. Ingram

**Affiliations:** ^1^Division of Food Processing Science and Technology, U. S. Food and Drug Administration, Bedford Park, IL, United States; ^2^Division of Produce Safety, U. S. Food and Drug Administration, College Park, MD, United States

**Keywords:** enoki mushrooms, heat treatment, *Listeria*, ramen, *Salmonella*, wood ear mushrooms

## Abstract

Recent listeriosis and salmonellosis outbreaks in the U.S. have been associated with consumption of specialty mushrooms, including enoki and wood ear. These mushrooms are commonly consumed in Asian dishes such as ramen noodle soup and are often used as raw garnishes. No current guidelines exist for the serving temperature of ramen broth in restaurants for safety. The objective of this study was to evaluate the inactivation of both *Listeria monocytogenes* and *Salmonella enterica* on enoki and wood ear mushrooms based on the ramen broth temperature. Fresh mushrooms were chopped into pieces, inoculated with four-strain cocktails of *L. monocytogenes* or *S. enterica*, dried at ambient conditions for 30 min, then placed into bowls. Ramen broth (i.e., pork bone broth, Tonkotsu) at initial temperatures of 60, 70, 80, 90, or 100°C was poured over the mushrooms, submerging them. The bowls were held at ambient conditions while the broth temperatures were monitored, and mushrooms were sampled at intervals up to 60 min. From the broth temperature profiles and the pathogen log reductions of each mushroom type, the non-isothermal log-linear model was used to obtain maximum inactivation rates. The maximum pathogen inactivation on both mushroom types occurred within the first 5 min, regardless of the initial broth temperature. Broth temperatures of 60 and 70°C resulted in reductions of only 1–3 log CFU/g (inactivation rates of 0.33–4.72 log CFU/g/min), while reductions of 4–5 log CFU/g (inactivation rates of 8.74–17.21 log CFU/g/min) were observed with 80°C broth. The use of 90 and 100°C broth resulted in higher reductions of >5 log CFU/g (inactivation rates of 8.56–28.08 log CFU/g/min). In all cases, surviving populations, often >2.4 log CFU/g, were observed after 60 min. The results from this study can assist in the development of guidelines on the safe serving temperature of ramen soup at restaurants.

## 1 Introduction

Specialty mushrooms have been the subject of foodborne illness outbreaks, recalls, and import alerts in the United States in recent years (CDC, 2020b,a; FDA, 2020c,a, 2022c,d; CDC, 2023; FDA, 2023b). Firstly, two multistate listeriosis outbreaks linked to fresh enoki mushrooms occurred in 2020 and 2023, cumulatively resulting in 41 cases, 36 hospitalizations and four deaths (CDC, 2020a; FDA, 2020a; CDC, 2023). Epidemiological investigation during the 2020 outbreak revealed that 55% of ill individuals reported eating mushrooms, including enoki, in the month prior to illness onset (CDC, 2020a); investigation during the 2023 outbreak determined that two ill individuals reported eating enoki mushrooms or eating at restaurants with menu items containing enoki mushrooms (CDC, 2023). Secondly, a multistate salmonellosis outbreak associated with dried wood ear mushrooms occurred in 2020 and resulted in 55 cases and six hospitalizations (CDC, 2020b; FDA, 2020c). The dried wood ear mushrooms were sold in bulk to restaurants, and not available directly to consumers. The epidemiological investigation revealed that 22 of 23 interviewed ill individuals reported eating ramen at a restaurant in the week before illness onset, and 10 of the individuals reported eating wood ear mushrooms or ramen containing these mushroom (CDC, 2020b).

During and after the enoki and wood ear mushroom outbreaks, the U.S. Centers for Disease Control and Prevention (CDC) disseminated advice to both consumers and restaurants on their website on the safe handling and preparation of these food commodities (CDC, 2020a,b, 2023). For enoki mushrooms, the CDC advised that consumers should cook these mushrooms thoroughly prior to consumption and keep raw enoki separate from other foods which would be eaten without cooking. The CDC also advised restaurants to not serve raw enoki, to cook enoki thoroughly before serving to customers, to not use raw enoki as garnish, and to not add raw enoki on top of soup dishes right before serving as the mushrooms would not get hot enough to kill *Listeria*. For dried wood ear mushrooms, the CDC advised that in general, dried mushrooms should always be rehydrated using boiling water to kill any pathogens.

Enoki and wood ear mushrooms are often used as ingredients or as toppings or garnishes on Asian dishes including stir-fry, stew, and ramen soup. Ramen is a popular food that generally consists of broth, noodles, cooked meat, and other cooked vegetables, as well as raw garnishes often including mushrooms, green onions, and bean sprouts. Although the ramen soup broth is served hot, the raw garnishes would likely not be cooked or only partially cooked by the broth. Therefore, bacterial pathogens on the garnishes, if present, would not be inactivated. A recent study surveying Asian restaurants in the U.S. on their handling practices and risk perceptions of dried wood ear mushrooms determined that various preparation practices (i.e., rehydration, blanching) and storage conditions were used for these mushrooms prior to adding to dishes (Chen et al., 2024). Participants in the study used a wide range of rehydration temperatures for the dried mushrooms, with only 12% using boiling water as suggested by the CDC. Only a few participants who did not use boiling water indicated that they blanched the wood ear mushrooms prior to incorporation into dishes. One of the interviewed restaurants indicated that they used cold water for rehydration and did not blanch the mushrooms prior to adding as a garnish on ramen.

In the recent outbreaks associated with specialty mushrooms, some ill individuals reported eating ramen soup, possibly containing raw enoki or dried wood ear mushrooms which had been rehydrated. It is not known what the serving temperature of the ramen broth was, what the mushroom preparation practices were, or if consumers submerged the mushrooms in the hot broth prior to consumption (Chen et al., 2024). No guidelines are currently available for the serving temperature of ramen soup broth in restaurants for safety. While it is recommended that cooked foods be heated to 57°C prior to consumption for safety (FDA, 2022b), this temperature of ramen soup broth may be ineffective at inactivating pathogens on mushrooms used as garnishes, as previous research has indicated that hot-air at 55 or 60°C resulted in minimal log reductions of *L. monocytogenes* and *S. enterica* on mushrooms (Kragh et al., 2022; Salazar et al., 2023). The objective of this study was to utilize predictive modeling to evaluate the inactivation of both *L. monocytogenes* and *S. enterica* on enoki and wood ear mushrooms used as garnishes on ramen soup based on the broth serving temperature. The results from this study can assist in the development of guidelines on the safe serving temperature of ramen soup at restaurants.

## 2 Materials and methods

### 2.1 Strains and culture conditions

A four-strain cocktail of either *Listeria monocytogenes* or *Salmonella enterica* was used in this study. For *L. monocytogenes*, the strains included LS806 (isolated from hummus), LS3132 (isolated from avocado), LS1863 (FDA1142659-C001-001, enoki mushroom outbreak isolate), and Scott A [clinical isolate (Briers et al., 2011)]. For *S. enterica*, the strains included Enteritidis PT30 (ATCC BAA-1045, almonds isolate), Agona [447967, roasted oats cereal isolate (CDC, 1998)], Alachua [CFSAN107331, peach leaf isolate (FDA, 2020b)], and Poona 8785 (CFSAN038692, cucumber isolate). All strains were rifampicin resistant (100 μg/mL). Each strain was cultured in Tryptic Soy Broth (TSB; Becton, Dickinson and Co., Sparks, MD, USA) for 16–18 h at 37°C. One hundred μL of each culture was plated onto Tryptic Soy Agar (TSA; Becton, Dickinson and Co.) and incubated at 37°C for 24 h. Cells were harvested from agar plates using a sterile spreader and 1 mL Butterfields’s Phosphate Buffer (BPB, pH 7.2). Harvested cells were combined in equal volumes to create a four-strain cocktail of either *L. monocytogenes* or *S. enterica* of approximately 10 log CFU/mL. To verify the initial population levels, both cocktails were serially diluted in BPB and plated onto Brain Heart Infusion Agar (BHIA; Becton, Dickinson and Co.). Agar plates were incubated at 37°C for 24–48 h prior to enumeration.

### 2.2 Mushroom preparation and inoculation

Fresh raw enoki and wood ear mushrooms were obtained from local retail grocers (IL, USA) and stored at 5°C for up to 24 h before use. The bottom 3 cm of the enoki mushrooms were discarded, and the enoki were chopped into 2.5–3.0 cm long pieces. Wood ear mushrooms were chopped into 2.5–3.0 cm long × 0.5–1.0 cm wide pieces. Individual sterile knives and cutting boards were used. Mushroom pieces were portioned into 10-cm foil pans of 10 g each. Each 10-g portion was inoculated with 100 μL of the *L. monocytogenes* or *S. enterica* cocktail (10 dots of 10 μL each distributed across the pieces). Inoculated mushrooms were dried for 30 min in a biosafety cabinet with the blower on. After drying, mushrooms were placed into heat-resistant mesh tea bags with drawstrings (8 cm × 10 cm) for ease of sampling.

### 2.3 Ramen soup broth preparation

Concentrated pork bone broth (i.e., Tonkotsu ramen broth) was purchased from a wholesale restaurant supply store and stored unopened at room temperature prior to use. Concentrated broth was diluted with water according to the manufacturer’s instructions to prepare 6 L in an 8-Qt (7.6 L) stainless steel Bain Marie pot. The pots were covered with foil and autoclaved for 30 min at 121°C (with a resulting temperature of approximately 95°C). For required broth temperatures of 60, 70, 80, or 90°C, broth was tempered in a water bath until the desired temperature was reached (±0.5°C). For a required broth temperature of 100°C, broth was stirred and heated on a hot plate to raise the temperature to 100°C (±0.5°C). A large 500-mL capacity sterile stainless steel ladle was added to the hot broth (as to not introduce a cold ladle into the broth prior to inactivation trials). Throughout tempering or heating, the broth temperature was measured using a digital data logging thermometer with dual input thermocouples (Fluke 52 II B, Fluke Corporation, Everett, WA, USA).

### 2.4 Treatment of mushrooms with ramen broth

Plastic ramen soup bowls (1-L capacity) were used for the experiments. Twelve bowls were used for each trial (six for enoki and six for wood ear). Each bowl corresponded to a different length of time (1, 3, 5, 15, 30, and 60 min). To each sterile bowl, triplicate enoki or wood ear mushroom samples were added. Once the broth was tempered or heated to the desired temperature, 500 mL of broth was added on top of the mushrooms in each bowl using the ladle, ensuring that mushrooms were submerged in the broth. Bowls were allowed to stand at room temperature (22–23°C) throughout the trial and the broth temperature was measured every min using a digital data logging thermometer. After 1, 3, 5, 15, 30, or 60 min, the triplicate mushrooms samples were removed from the broth and placed into stomacher bags containing 90 mL cold BPB for 60, 70, 80, and 90°C trials or 90 mL of cold Buffered *Listeria* Enrichment Broth (BLEB) or cold Buffered Peptone Water (BPW) for 100°C trials (for enrichment of *L. monocytogenes* or *S. enterica*, respectively, if required). Bags containing BPB, BLEB, or BPW were stored at 5°C and removed immediately before use. Four to six trials of each mushroom, pathogen, and broth temperature combination were conducted.

### 2.5 Enumeration and enrichment of *L. monocytogenes* and *S. enterica*

Stomacher bags containing mushrooms and 90 mL of BPB, BLEB, or BPW, were mixed for 1 min in a stomacher at 150 rpm (Stomacher 400 Circulator, Seward, U.K.). Homogenates were serially diluted and plated onto BHIA supplemented with 100 μg/mL of rifampicin (Fisher Scientific, Waltham, MA, USA) (BHIA^rif^) for enumeration. For trials conducted at 100°C, enrichment of *L. monocytogenes* and *S. enterica* was also conducted since populations were expected to be lower than the limit of enumeration of the plate count assay (2.30 log CFU/g). Enrichment was conducted as per the Bacteriological Analytical Manual (FDA, 2022a, 2023a) using specified confirmation agars as well as BHIA^rif^. All agar plates were incubated at 37°C for 24–48 h.

### 2.6 Pathogen inactivation modeling

The logged temperature data for each independent trial was modeled during cooling. The data indicated first order cooling, given by Equation 1:


(1) $$\ln\frac{T-T_{f}}{T_{0}-T_{f}}=-\frac{t}{\tau }$$

where *T*_0_ is the zero-time temperature, *T_f_* is the final temperature, and *τ* is the time constant. The time constant, *τ*, was determined by fitting Equation 1 to the data after 3 min. The broth temperatures from the first 3 min of each trial were then back calculated, including *T_0_*, which was subsequently used to determine the maximum rate of pathogen inactivation.

A log-linear model (Bigelow and Esty, 1920) was fit to populations (log CFU/g) of *L. monocytogenes* and *S. enterica* on mushrooms from 0 to 60 min using the Solver add-in in Excel. The isothermal log-linear model for inactivation in the form using *D* and *z* values is presented in Equation 2:


(2) $$\log\frac{N_{0}}{N}=\frac{t}{D_{T_{ref}}}10^{\frac{T-T_{ref}}{z}}$$

where *N* is the population at time *t*, *N*_0_ is the initial population, $D_{T_{ref}}$ is the decimal reduction time at temperature *T*_*ref*_, *T* is the temperature at which inactivation occurs, and *z* is the *z* value. *T_ref_* was set to 70°C.

As written, Equation 2 includes an assumption of constant temperature during the inactivation time. However, the pathogen inactivation in this study occurred while temperature was changing as the broth cooled. Therefore, the inactivation is given by Equation 3:


(3) $$\log\frac{N_{0}}{N}=\frac{1}{D_{T_{ref}}}\int_{0}^{t}10^{\left(T\left(\theta \right)-T_{ref}\right)/z}d\theta$$

where *θ* is the integration variable representing time, and *T(θ)* is the variation in temperature over the inactivation time. This integral was easily accomplished in Excel by arranging the times and corresponding temperatures and calculated pathogen reductions in columns next to each other. Since temperature values were logged every min, a total of 61 rows of data were generated. Integration was carried out using the trapezoidal rule where the pathogen reduction during each min was calculated by Equation 4:


(4) $$\log\frac{N_{i-1}}{N_{i}}=\frac{t_{i}-t_{i-1}}{D_{T_{ref}}}\frac{10^{\left(T_{i-1}-T_{ref}\right)/z}+10^{\left(T_{i}-T_{ref}\right)/z}}{2}$$

where *i* is the *i*th row of data and *i* − 1 is the row directly above it. The term, $t_{i}-t_{i-1}$, is the time interval between each row, which is always 1 min in this study. Equation 4 represents an incremental reduction for each row. If each is added to the result from the row above, a running total of reduction is obtained at each time point. Reduction data was then compared to those calculated at the 6 timepoints by subtracting and squaring their difference. The squared differences were summed, and the minimization of this sum was the objective of Excel’s Solver routine. Minimization was obtained by adjusting the values of $D_{T_{ref}}$ and *z* and, when accomplished, gave the values for $D_{T_{ref}}$ and *z* that best fit the calculated reductions to the reduction data. To carry out the fitting, initial guesses for $D_{T_{ref}}$ and *z* were made, using the sum of the squared differences as a guide to obtain a reasonable starting point.

Since the maximum inactivation is usually given as *k*_max_ when inactivation is of the form in Equation 5:


(5) $$\ln\frac{N}{N_{0}}=kt$$

the conversion from *D* and *z* values to *k*_max_ is given by Equation 6:


(6) $$k_{\text{max}}=\frac{\ln10}{D_{T_{ref}}}10^{\frac{T_{0}-T_{ref}}{z}}$$

### 2.7 Secondary modeling

To understand the relationship of the inactivation rate (*k*_max_, log CFU/g/min) as a function of the ramen broth temperature, the inactivation rate of each pathogen on both mushroom types or on enoki or wood ear separately from each trial was plotted against the zero-time broth temperature once poured into bowls (*T_0_*). The mean inactivation rate from all trials for *L. monocytogenes* or *S. enterica* on both mushroom types were also plotted against the initial broth temperature (*T_i_*). To assess linearity, linear regression was used and the goodness of fit, *r*^2^, was reported.

### 2.8 Statistical analyses

Differences in *L. monocytogenes* or *S. enterica* populations on mushrooms during submersion in ramen broth were statistically analyzed using ANOVA with Tukey’s post-hoc test (*α* = 0.05). Differences in mean inactivation rates [*k*_max_ (log CFU/g/min)] between different initial broth temperatures (*T_i_*) for the same pathogen on the same mushroom type and also between mushroom types at the same initial broth temperature were statistically compared using ANCOVA with Tukey’s post-hoc test (*α* = 0.05). Root mean square error (RMSE) was reported for each *k*_max_ value obtained for each trial.

## 3 Results

### 3.1 Ramen broth temperature profiles

The temperature data from the ramen broth cooling generally exhibited a first order cooling rate as described in Equation 1. However, the pouring of the broth into the bowls introduced variation in the initial recorded temperature due to the arbitrary placement of the thermocouples and the purposeful variability of pouring broth into the bowls. By fitting a first order cooling rate to the longer-term data, all of which showed first-order cooling, then back-calculating to the initial temperature, a more consistent initial temperature was obtained. One such set of data is shown in Supplementary Figure S1. In this example, although the data showed an initial temperature of 52°C, the cooling data indicated that for this particular bowl, a more reasonable initial temperature was 57°C.

When calculating reductions to eventually establish pathogen inactivation rates (*k*_max_), the *D* and *z* version of first order kinetics, Equation 2, was used instead of the form shown in Equation 5. In that form, the temperature dependence of *k* is given by Equation 7:


(7) $$k=k_{0}e^{-\frac{E}{RT}}$$

where *k_0_* and *E/R* are roughly analogous to *D* and *z*. When using this form, Excel’s Solver add-in could not converge onto values of *k_0_* and *E/R* that minimized the difference between measured data and calculated data. However, Solver did converge using Equation 2, thereby obtaining *D* and *z* values. Equation 6 was then used to obtain *k*_max_. Supplementary Figure S2 displays an example fit for a trial at an initial broth temperature (*T_i_*) of 80°C.

The temperature profiles of the ramen broth at the initial temperatures (*T_i_*) of 60, 70, 80, 90, or 100°C when poured into bowls and kept at room temperature for 60 min are presented in Supplementary Figures S3, S4 for each *L. monocytogenes* and *S. enterica* trial, respectively. For each initial broth temperature, the zero-time temperatures of the broth once poured into the bowls (*T_0_*) for each independent trial are presented in Tables 1, 2, for each *L. monocytogenes* and *S. enterica* trial, respectively. At an initial broth temperature of 60°C, for example, the temperature of the broth once poured into the bowls ranged from 49.19 to 60.10°C, with a mean of 53.72°C (*n* = 12). In two trials, this temperature was >10°C lower than the initial broth temperature. In another experiment, at an initial broth temperature of 70°C, the temperature of the broth once poured into the bowls ranged from 51.69 to 59.40°C, with a mean of 55.28°C (*n* = 10). In this experiment the temperature of the broth once poured into the bowls was always >10°C lower than the initial broth temperature.

**Table 1:** Kinetic parameters of the dynamic log-linear model to describe the inactivation of *Listeria monocytogenes* on enoki and wood ear mushrooms based on the ramen broth temperature.

*T_i_* (°C)^a^	Mushroom type	*T_0_* (°C)^b^	*k*_max_^c^ (log CFU/g/min)	RMSE^d^	Mean *k*_max_^e^ (log CFU/g/min ± SE^f^)
60	Enoki	49.19	0.33	0.1585	2.31 ± 0.72^aA^
		53.55	1.98	0.2557	
		59.51	4.62	0.1774	
	Wood ear	50.21	1.16	0.2719	0.66 ± 0.19^aB^
		51.34	0.05	0.0271	
		57.15	0.76	0.1202	
70	Enoki	51.69	2.26	0.219	2.45 ± 0.53^aA^
		54.63	1.00	0.2899	
		55.44	4.09	0.4848	
	Wood ear	52.11	1.93	0.2019	2.51 ± 0.26^bA^
		56.11	2.22	0.3352	
		59.40	3.40	0.4036	
80	Enoki	61.50	12.15	0.8629	11.35 ± 0.55^bA^
		67.77	10.59	0.5250	
	Wood ear	66.31	17.21	0.8293	15.06 ± 1.52^cB^
		68.05	12.91	0.4900	
90	Enoki	71.05	28.08	0.1313	22.34 ± 0.56^cA^
		76.08	18.89	0.4238	
	Wood ear	65.38	21.54	0.4050	23.49 ± 3.25^dA^
		71.80	23.13	0.3341	
100	Enoki	74.85	25.49	0.1987	24.50 ± 0.47^cA^
		78.50	25.12	0.1405	
		78.93	22.89	0.0367	
	Wood ear	76.35	18.79	0.0060	22.83 ± 1.33^dA^
		78.52	26.78	0.1172	
		79.29	22.93	0.2140	

^a^*T_i_*, initial temperature of the ramen broth; ^b^*T_0_*, temperature of the broth once poured into the bowls; ^c^*k*_max_, inactivation rate (log CFU/g/min), each value represents an individual trial; ^d^root mean square error; ^e^mean *k*_max_ from 2 to 3 individual trials at the same *T_i_*; ^f^SE, standard error. Mean *k*_max_ values with different lowercase letters are significantly different for the same mushroom type between different *T_i_*. Mean *k*_max_ values with different uppercase letters are significantly different between different mushroom types at the same *T_i_*.

**Table 2:** Kinetic parameters of the dynamic log-linear model to describe the inactivation of *Salmonella enterica* on enoki and wood ear mushrooms based on the ramen broth temperature.

*T_i_* (°C)^a^	Mushroom type	*T_0_* (°C)^b^	*k*_max_^c^ (log CFU/g/min)	RMSE^d^	Mean *k*_max_^e^ (log CFU/g/min ± SE^f^)
60	Enoki	50.58	2.04	0.1118	2.68 ± 0.49^aA^
		51.67	4.37	0.8833	
		60.10	1.63	0.0790	
	Wood ear	49.95	1.45	0.0592	1.07 ± 0.11^aB^
		52.30	0.78	0.1415	
		59.09	0.99	0.0823	
70	Enoki	55.46	2.74	0.4214	3.73 ± 0.70^aA^
		57.63	4.72	0.1872	
	Wood ear	52.38	2.50	0.2617	4.42 ± 1.36^bA^
		57.92	6.34	0.2996	
80	Enoki	67.34	13.99	0.6170	11.98 ± 0.94^bA^
		65.09	8.74	0.4887	
		68.02	13.21	0.4080	
	Wood ear	63.59	15.23	0.4824	15.08 ± 0.14^cB^
		64.13	14.61	0.5326	
		67.18	15.39	0.3962	
90	Enoki	71.95	24.04	0.2822	18.61 ± 3.84^cA^
		78.54	13.18	0.3948	
	Wood ear	68.74	13.32	0.8319	18.00 ± 3.31^dA^
		70.83	22.68	0.6227	
100	Enoki	74.03	8.56	0.4442	17.06 ± 2.46^cA^
		75.68	21.78	0.4121	
		79.43	20.82	0.4678	
	Wood ear	73.50	24.04	0.0953	17.03 ± 2.10^dA^
		78.52	15.20	0.6232	
		82.50	11.86	0.6063	

^a^*T_i_*, initial temperature of the ramen broth; ^b^*T_0_*, temperature of the broth once poured into the bowls; ^c^*k*_max_, inactivation rate (log CFU/g/min), each value represents an individual trial; ^d^root mean square error; ^e^mean *k*_max_ from 2 to 3 individual trials at the same *T_i_*; ^f^SE, standard error. Mean *k*_max_ values with different lowercase letters are significantly different for the same mushroom type between different *T_i_*. Mean *k*_max_ values with different uppercase letters are significantly different between different mushroom types at the same *T_i_*.

At an initial broth temperature of 80°C, the temperature of the broth once poured into the bowls ranged from 61.50 to 68.05°C, with a mean of 65.90°C (*n* = 10). The temperature of the broth once poured into the bowls was always >11°C lower than the initial broth temperature. At an initial broth temperature of 90°C, the temperature of the broth once poured into the bowls ranged from 65.38 to 78.54°C, with a mean of 71.80°C (*n* = 8). In two trials, the temperature of broth once poured into the bowls was >20°C lower than the initial broth temperature. At an initial broth temperature of 100°C, the temperature of the broth once poured into the bowls ranged from 74.03 to 82.50°C, with a mean of 77.87°C (*n* = 11). In two trials, the temperature of broth once poured into the bowls was >25°C lower than the initial broth temperature.

The temperature of the broth in the bowls at 30 min ranged from 33.25 to 39.90°C for broth at an initial temperature of 60°C; from 39.50 to 43.40°C for broth at an initial temperature of 100°C. At 60 min, the temperature of the broth in the bowls ranged from 27.06 to 30.70°C for broth at an initial temperature of 60°C; and from 30.80 to 33.50°C for broth at an initial temperature of 100°C.

### 3.2 Pathogen population dynamics on mushroom garnishes

**Figure 1:**
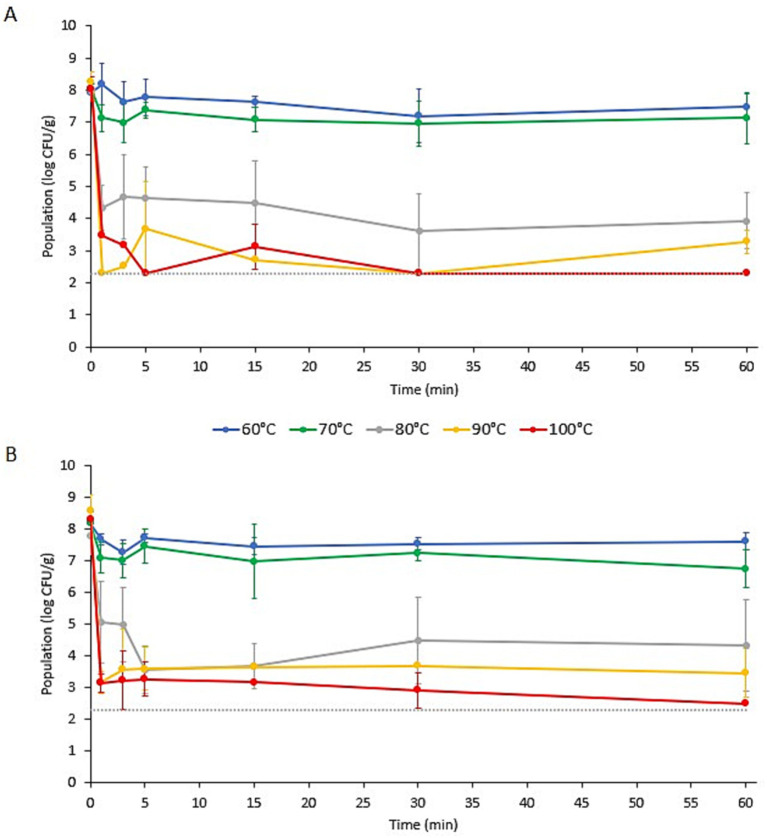
Populations of *Listeria monocytogenes* on **(A)** wood ear and **(B)** enoki mushroom garnishes in ramen broth with an initial temperature (*T_i_*) of 60, 70, 80, 90, or 100°C. Data are means ± standard deviations (*n* = 6 or 9). Dotted line indicates the lower limit of enumeration (2.30 log CFU/g).

**Figure 2:**
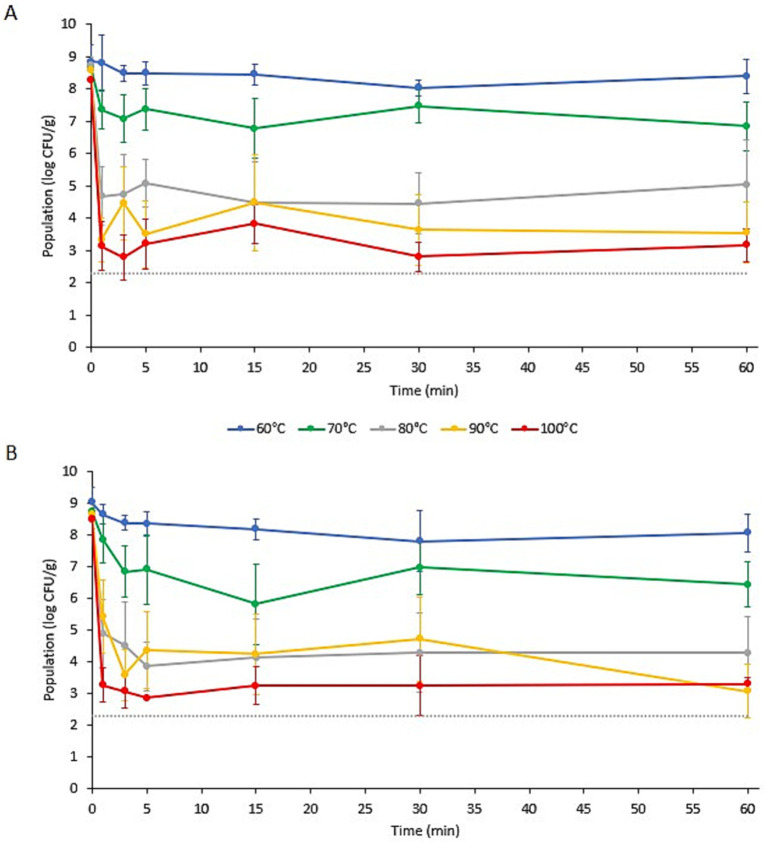
Populations of *Salmonella enterica* on **(A)** wood ear and **(B)** enoki mushroom garnishes in ramen broth with an initial temperature (*T_i_*) of 60, 70, 80, 90, or 100°C. Data are means ± standard deviations (*n* = 6 or 9). Dotted line indicates the lower limit of enumeration (2.30 log CFU/g).

The populations of *L. monocytogenes* and *S. enterica* on the specialty mushroom garnishes during submersion in ramen broth at different initial temperatures (*T_i_*) are presented in Figures 1, 2, respectively. Prior to submersion, the initial populations of *L. monocytogenes* and *S. enterica* on the two mushroom types were not significantly different. The populations of *L. monocytogenes* on the enoki and wood ear ranged from 7.78 ± 0.58 to 8.57 ± 0.51 log CFU/g and 7.93 ± 0.14–8.28 ± 0.30 log CFU/g, respectively. The populations of *S. enterica* on the enoki and wood ear ranged from 8.47 ± 0.41 to 9.03 ± 0.47 log CFU/g and 8.25 ± 0.36–8.84 ± 0.50 log CFU/g, respectively. When an initial broth temperature of 60°C (*T_i_*) was used, only minimal population reductions were observed. The greatest reduction of *L. monocytogenes* occurred on enoki after 3 min (0.93 log CFU/g) and on wood ear after 30 min (0.73 log CFU/g). The greatest reduction in *S. enterica* occurred after 30 min on both mushroom types: 1.23 and 0.82 log CFU/g reductions on enoki on wood ear, respectively. With the exception of *L. monocytogenes* on wood ear, significantly higher population reductions were observed when broth at an initial temperature of 70°C (*T_i_*) was used. The greatest reduction of *L. monocytogenes* occurred on enoki after 60 min (1.45 log CFU/g) and on wood ear after 30 min (1.07 log CFU/g). The greatest reduction in *S. enterica* occurred after 15 min on both mushroom types: 2.93 and 1.92 log CFU/g reductions on enoki on wood ear, respectively.

When an initial broth temperature of 80, 90, or 100°C (*T_i_*) was used, more substantial log reductions were observed for both pathogens on both mushroom types. When broth at an initial temperature of 80°C (*T_i_*) was used, log reductions after only 1 min ranged from 2.71 log CFU/g (for *L. monocytogenes* on enoki) to 4.05 log CFU/g (for *S. enterica* on wood ear). On enoki, the greatest reduction of both pathogens occurred after 5 min (4.21 and 4.62 log CFU/g for *L. monocytogenes* and *S. enterica*, respectively); for wood ear, the greatest reduction occurred after 30 min (4.40 and 4.27 log CFU/g, respectively). When broth at an initial temperature of 90°C (*T_i_*) was used, the population of *L. monocytogenes* on wood ear was below the level of enumeration (<2.30 log CFU/g) after only 1 min, corresponding to a reduction of >5.98 log CFU/g. In the same amount of time, the reduction of *L. monocytogenes* on enoki was 5.42 log CFU/g, while *S. enterica* was reduced by 3.23 and 5.21 log CFU/g on enoki and wood ear, respectively. After the initial reduction at 1 min, both pathogens survived for the 60 min duration in the broth, with no significant further decrease in population.

When broth at an initial temperature of 100°C (*T_i_*) was used, the populations of *L. monocytogenes* were reduced by 5.15 and 4.59 log CFU/g on enoki and wood ear, respectively, after 1 min. In the same amount of time, *S. enterica* was reduced by 5.24 and 5.11 log CFU/g on enoki and wood ear, respectively. After the initial reduction at 1 min, no further population reduction was observed for *L. monocytogenes* on enoki or for *S. enterica* on either mushroom type. However, the *L. monocytogenes* population on wood ear was significantly further reduced at 5 min to <2.30 log CFU/g, corresponding to a reduction of >5.76 log CFU/g. *L. monocytogenes* was also <2.30 log CFU/g after 30 and 60 min, however, the pathogen was still detected via enrichment in all samples at each timepoint (*n* = 9). It is noted that surviving populations of both pathogens remained on both mushroom types after 60 min regardless of the initial broth temperature used.

### 3.3 Pathogen inactivation rates on mushroom garnishes

The inactivation rates (*k*_max_, log CFU/g/min) for each pathogen on the enoki and wood ear mushrooms for each individual trial at each initial broth temperature (*T_i_*) are presented in Tables 1, 2, for *L. monocytogenes* and *S. enterica*, respectively. Mean inactivation rates for each pathogen on each mushroom type at each initial broth temperature are also presented. Generally, inactivation rates for both pathogens on the mushrooms increased with increasing initial broth temperature; however, some exceptions occurred, especially with 60 and 70°C, where the temperature of the broth once poured into the bowls (*T*_0_) tended to overlap. When broth at an initial temperature of 60°C (*T_i_*) was used, inactivation rates for the individual trials ranged from 0.05 log CFU/g/min (for *L. monocytogenes* on wood ear) to 4.26 log CFU/g/min (for *L. monocytogenes* on enoki). The mean inactivation rates for both *L. monocytogenes* and *S. enterica* were significantly higher on enoki (2.31 and 2.68 log CFU/g/min, respectively) compared to wood ear (0.66 and 1.07 log CFU/g/min, respectively). When broth at an initial temperature of 70°C (*T_i_*) was used, inactivation rates for the individual trials ranged from 1.00 log CFU/g/min (for *L. monocytogenes* on enoki) to 6.34 log CFU/g/min (for *S. enterica* on wood ear). There was no significant difference between the mean inactivation rates for *L. monocytogenes* and *S. enterica* on either mushroom.

When broth at an initial temperature of 80°C (*T_i_*) was used, inactivation rates for the individual trials ranged from 8.74 log CFU/g/min (for *S. enterica* on enoki) to 17.21 log CFU/g/min (for *L. monocytogenes* on wood ear). The mean inactivation rates for both *L. monocytogenes* and *S. enterica* were significantly higher on wood ear (15.06 and 15.08 log CFU/g/min, respectively) compared to enoki (11.35 and 11.98 log CFU/g/min, respectively). When broth at an initial temperature of 90°C (*T_i_*) was used, inactivation rates for the individual trials ranged from 13.18 log CFU/g/min (for *S. enterica* on enoki) to 28.08 log CFU/g/min (for *L. monocytogenes* on enoki). There was no significant difference between the mean inactivation rates for *L. monocytogenes* and *S. enterica* on either mushroom. When broth at an initial temperature of 100°C (*T_i_*) was used, inactivation rates for the individual trials ranged from 8.56 log CFU/g/min (for *S. enterica* on enoki) to 26.78 log CFU/g/min (for *L. monocytogenes* on wood ear). The mean inactivation rates for *L. monocytogenes* on enoki and wood ear (24.50 and 22.84 log CFU/g/min, respectively) were significantly higher than those for *S. enterica* (17.06 and 17.03 log CFU/g/min, respectively).

### 3.4 Relationship between pathogen inactivation rates and broth temperature

**Figure 3:**
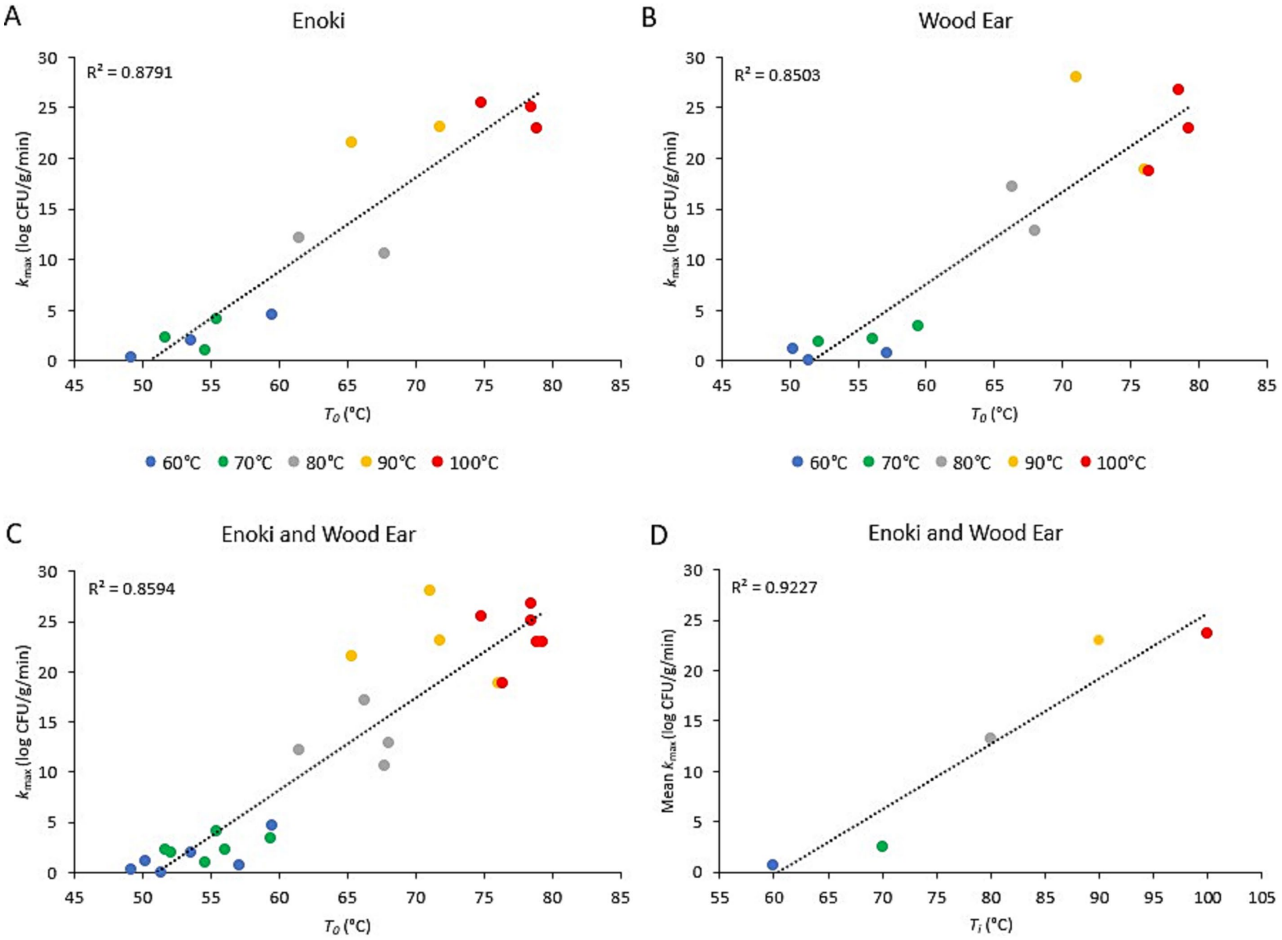
Relationship between the inactivation rate of *Listeria monocytogenes* on enoki and wood ear mushroom garnishes and the ramen broth temperature. Inactivation rate (*k*_max_) on **(A)** enoki, **(B)** wood ear, and **(C)** both mushrooms versus time-zero temperature of ramen broth in bowls (*T_0_*). Each data point represents an individual trial. **(D)** Mean inactivation rate (*k*_max_) versus ramen broth initial temperature (*T_i_*). Each data point represents the mean of 4–6 individual trials. Dotted lines represent linear regression.

**Figure 4:**
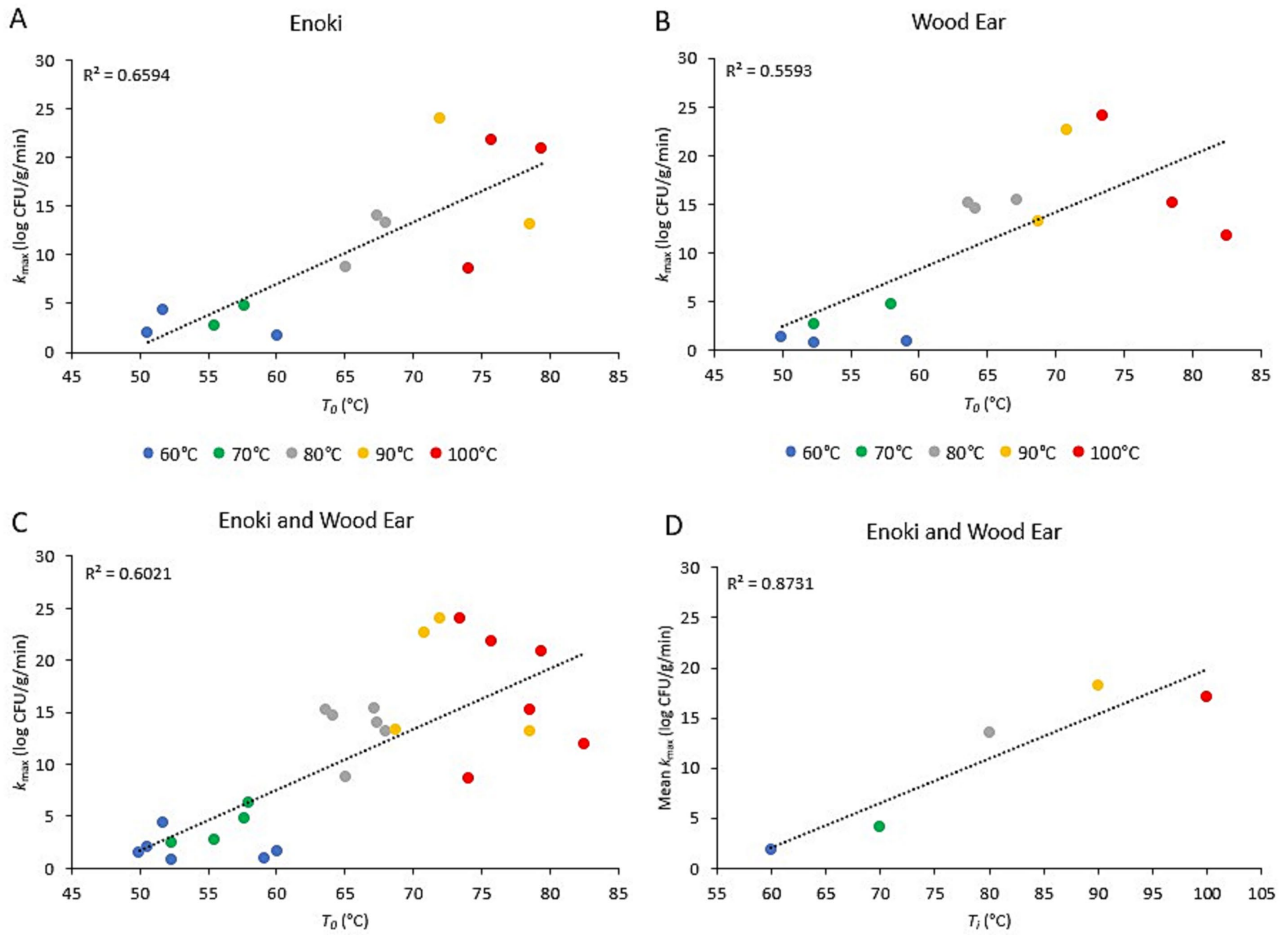
Relationship between the inactivation rate of *Salmonella enterica* on enoki and wood ear mushroom garnishes and the ramen broth temperature. Inactivation rate (*k*_max_) on **(A)** enoki, **(B)** wood ear, and **(C)** both mushrooms versus time-zero temperature of ramen broth in bowls (*T_0_*). Each data point represents an individual trial. **(D)** Mean inactivation rate (*k*_max_) versus ramen broth initial temperature (*T*_i_). Each data point represents the mean of 4–6 individual trials. Dotted lines represent linear regression.

The relationship between the inactivation rates of both pathogens on the two mushroom types and the initial broth temperature (*T_i_*) or the temperature of the broth once poured into the bowls (*T_0_*) is presented in Figures 3, 4, for *L. monocytogenes* and *S. enterica*, respectively. For *L. monocytogenes*, a linear relationship was observed between the inactivation rates on enoki, wood ear, and both mushroom types and the temperature of the broth once poured into the bowls (*T_0_*) (*r*^2^ = 0.88, 0.85, and 0.86, respectively). For *S. enterica*, a weaker linear relationship was observed for enoki (*r*^2^ = 0.66), wood ear (*r*^2^ = 0.56) and both mushroom types (*r*^2^ = 0.66, 0.56, and 0.60, respectively). For both pathogens, a linear relationship was observed between the mean inactivation rates for both mushroom types and the initial broth temperature (*T_i_*): *r*^2^ = 0.92 and 0.87 for *L. monocytogenes* and *S. enterica*, respectively.

## 4 Discussion

This study examined the inactivation of both *L. monocytogenes* and *S. enterica* on enoki and wood ear mushrooms in ramen broth based on the broth temperature. Ramen noodle soup, as well as other Asian dishes, are often accompanied by raw garnishes, either served on the side or on top of the broth. In the recent foodborne outbreaks associated with specialty mushrooms, some of the ill individuals reported eating ramen soup, which possibly contained enoki or wood ear mushrooms (CDC, 2020b, 2023). In the outbreaks, it is not known what the serving temperature of the ramen broth was or if consumers submerged the mushrooms in the hot broth, and for how long, prior to consumption (Chen et al., 2024). It is therefore essential to understand the population dynamics of these foodborne pathogens on these mushrooms when submerged in ramen broth initially heated to different temperatures.

Five different ramen broth temperatures were examined in this study ranging from 60 to 100°C to include a wide range of possible broth temperatures in restaurants. Once these broths were ladled into bowls containing mushroom samples, the broth temperature decreased by >11°C (in the case of trials using 80°C broth) to as much as 25°C (in the case of two trials when 100°C broth was used). This temperature decrease may be attributed to the arbitrary placement of the thermocouples in the bowls and the purposeful variability of pouring broth into the bowls. In addition, the mushrooms, as well as the bowls themselves, were at room temperature. If ramen broth was poured over warm or hot ingredients, such as noodles or chashu, it is possible that the broth temperature once poured into the bowls may be closer to the initial broth temperature. Therefore, the broth temperature profiles as reported in this study could be viewed as worst-case-scenarios when using only room temperature ingredients. Regardless of the initial broth temperature used in this study, the broth temperature decreased to 33–43°C after 30 min and to 27–33°C after 60 min, overall differences of only 10 and 7°C, respectively.

In general, the maximum pathogen inactivation on both mushroom types occurred within the first 5 min when completely submerged in the broth, regardless of the initial broth temperature used; these results can be viewed as a best-case scenario, as garnishes may not be completely submerged in the broth in a restaurant setting. In addition, a high initial pathogen inoculation level (i.e., 8–9 log CFU/g) was utilized in this study to evaluate inactivation rates, although it is possible that inactivation rates may differ if lower initial concentration are used. High levels of foodborne pathogens and *Enterobacteriaceae* have been identified on mushrooms. In response to the listeriosis outbreak in 2020, traceback and laboratory investigations determined that the implicated enoki mushrooms contained levels of *L. monocytogenes* as high as 5.90 log CFU/g (Pereira et al., 2023). High levels of *Enterobacteriaceae* have also been found on other types of mushrooms at retail markets, including wood ear, with populations as high as 6.5 log CFU/g (Venturini et al., 2011). In this study, broth temperatures of 60 and 70°C resulted in minimal reductions of only 1 and 1–2 log CFU/g, respectively (although a further 1 log CFU/g reduction in *S. enterica* occurred on enoki after 15 min with 70°C broth). Reductions of 4–5 log CFU/g for both pathogens were observed with 80°C broth, while higher reductions of >5 log CFU/g were observed with 90 and 100°C broth. It is noted that in all cases, surviving populations, often >2.4 log CFU/g, were observed from 15 to 60 min. While this study did not evaluate the possible detachment of pathogens from the mushrooms into the broth, the inactivation of pathogen population in the broth would most likely mimic the inactivation as observed on the mushrooms themselves.

Although not directly comparable, studies have assessed the fate of *L. monocytogenes* and *S. enterica* on mushrooms during isothermal dehydration in household food dehydrators at different temperatures (Kragh et al., 2022; Salazar et al., 2023). On enoki and wood ear mushrooms dehydrated at 90°C (air temperature 90.2°C), the populations of *L. monocytogenes* and *S. enterica* were reduced by >4 log CFU/g after 2–4 h; at 70 and 80°C (air temperatures 68.9 and 82.5°C, respectively), pathogens were reduced by >4 log CFU/g on wood ear after 4–8 h (Salazar et al., 2023). On portobello mushrooms, *L. monocytogenes* and *S. typhimurium* were reduced by only 2.5–2.6 log CFU/g after 8 h at 55°C (air temperature 46°C). Studies have also examined the heat resistance of *L. monocytogenes* on mushrooms during isothermal blanching at different temperatures (Doyle et al., 2001; Mazzotta, 2001). On mushrooms (variety unknown) treated with water at 60 or 62°C, the *D* values for *L. monocytogenes* were 0.69 and 0.30 min, respectively for stationary phase cells and 1.68 and 0.96 for starved cells (Mazzotta, 2001). The authors conclude that stressed cells are less heat resistant than their stationary counterparts on mushrooms. The inactivation results on mushrooms during blanching at 60 and 62°C are similar to those obtained in this study for *L. monocytogenes* and *S. enterica* at the initial broth temperature of 60°C, even though the broth in this study was left to cool naturally at room temperature.

Although this study utilized fresh specialty mushrooms, dried or rehydrated mushrooms are also used in ramen noodle soup and other Asian dishes. Instructions for the preparation and rehydration of dried mushrooms are generally not consistent between brands, however some brands suggest rehydrating dried wood ear mushrooms in warm water for 30 min. A recently published study examined the fate of *S.* Stanley on dried wood ear mushrooms during rehydration in warm water for 30 min (Patil et al., 2024). The study determined that the pathogen was reduced by 3.90 and >5 log CFU/g on the mushrooms when rehydrated with water at an initial temperature of 80°C (left to cool naturally at room temperature) after 2 and 10 min, respectively. Although the temperature profile of the water over time is not available, the water temperature most likely decreased more rapidly due to the smaller volume used (90 mL of water) compared to this study (500 mL of ramen broth). Regardless, the decrease in population of *S.* Stanley on dried wood ear mushroom rehydrated with 80°C water was comparable to the decrease of *S. enterica* on fresh wood ear mushroom observed in this study when ramen broth at an initial temperature of 80°C was used.

The pathogen inactivation results presented in this study are specific to the two specialty mushrooms examined, enoki and wood ear, the ramen broth used in this study (i.e., pork bone broth, Tonkatsu), and the use of high inoculation levels. This broth was chosen as a model due to its typical presence on the menus of ramen noodle restaurants. Future research could examine pathogen inactivation on different mushroom types or in different broth formulations. Results from this research provide information on the survival of *L. monocytogenes* and *S. enterica* on specialty mushrooms, commonly used as garnishes on ramen, when submerged in ramen broth at different initial temperatures. Results indicate that broth temperatures below 90°C may not be sufficient to reduce foodborne pathogens on mushrooms when fully submerged in broth and that higher broth temperatures should be utilized for safety.

## Data Availability

The original contributions presented in the study are included in the article/Supplementary material, further inquiries can be directed to the corresponding author.
