# Supplementary material for: Inactivation kinetics of Listeria monocytogenes and Salmonella enterica on specialty mushroom garnishes based on ramen soup broth temperature
Source: Front Microbiol. 2024 Dec 4;15:1485398. doi: 10.3389/fmicb.2024.1485398 (PMC11652501; doi:10.3389/fmicb.2024.1485398)

**Figure S1**. Comparison of logged temperature data and fitted values of the ramen broth during room temperature cooling using first order cooling, as depicted in Equation 1. The solid line indicates the logged temperature data while the dotted line indicates the fitted temperature data.

**Figure S2**. Comparisons of the observed *Salmonella enterica* reduction data to the fitted first order reduction equation, as depicted in Equation 3. Ramen broth was used at an initial temperature (*T_i_*) of 80°C.

**Figure S3**. Cooling temperature profiles of the ramen broth in bowls at room temperature (21-23°C) for 60 min when the initial temperatures of the broth (*T_i_*) were A) 60, B) 70, C) 80, D) 90, or E) 100°C. Each line represents an independent trial for *Listeria monocytogenes*; 4-6 trials were conducted for each initial temperature.


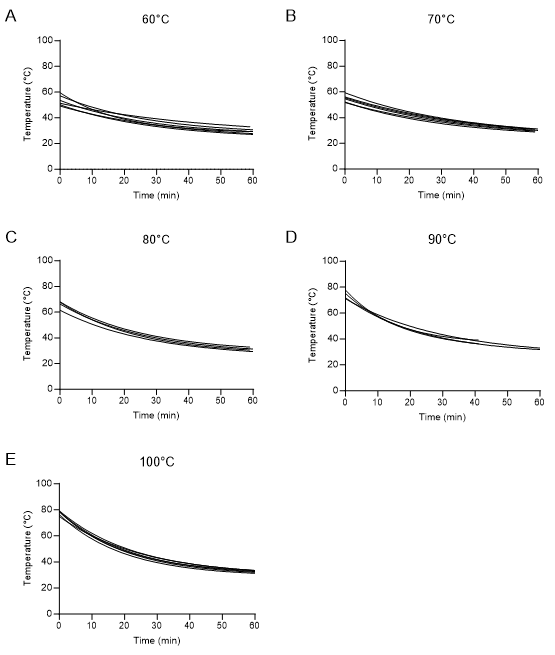


**Figure S4**. Cooling temperature profiles of the ramen broth at room temperature (21-23°C) for 60 min when the initial temperatures of the broth (*T_i_*) were A) 60, B) 70, C) 80, D) 90, or E) 100°C. Each line represents an independent trial for *Salmonella enterica*; 4-6 trials were conducted for each initial temperature.


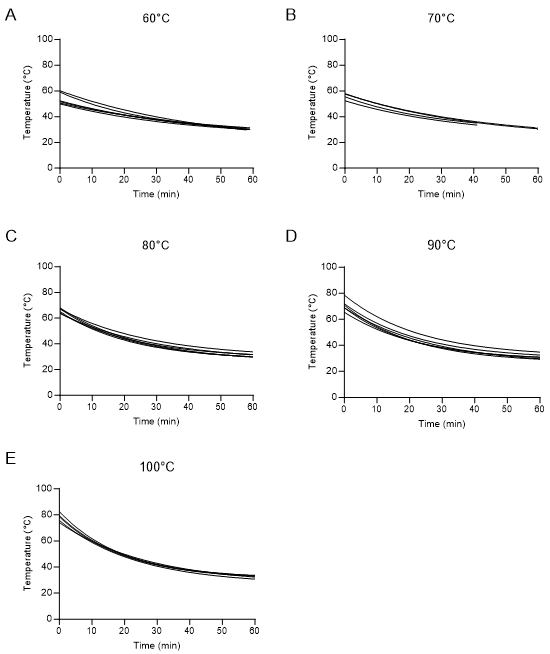

Supplement: Supplementary file 1 [file Table_1.docx]
